# Reflections on the Concomitants of the Restrictive Visitation Policy During the COVID-19 Pandemic: An Ubuntu Perspective

**DOI:** 10.3389/fsoc.2021.769199

**Published:** 2022-01-11

**Authors:** Fhumulani Mavis Mulaudzi, Rafiat Ajoke Anokwuru, Moselene A. R. Du-Plessis, Rachael T. Lebese

**Affiliations:** ^1^ Department of Nursing, University of Pretoria, Pretoria, South Africa; ^2^ Research Office, Faculty of Health Sciences, University of Venda, Thohoyandou, South Africa

**Keywords:** caregiving, Ubuntu perspective, restrictive visitation policy, COVID-19, reflections

## Abstract

Caregiving is a prominent concept in the Ubuntu philosophy, and caring and visitation of the sick is regarded as an example of Ubuntu. The restrictive visitation policy adopted in the hospitals during the coronavirus disease 2019 (COVID-19) pandemic affected the exhibition of this concept among patients, nurses, and families. The narrative inquiry was used to explore the reflections of the participants on the impact caused by the non-visitation policy experienced during the first and second waves of the COVID-19 pandemic in South Africa. The narrative inquiry approach allowed the participants to tell their story as it is unique to them. The study used purposive sampling technique to select five participants for the webinar. Three themes emerged from the narrated stories which are 1) moral anguish of the caregivers; 2) mental health instability, and 3) erosion of trust in health care practitioners (HCPs). The non-visitation hospital policy was intended to reduce the danger of spreading COVID-19 within and outside the hospital; however, the care provided was devoid of the values of Ubuntu such as mutual respect, relational, responsibility, reciprocity, and interconnectedness. In retrospect, a case-by-case application of the policy would reduce the non-desirable effect of the policy on the patients, nurses, and patients' family members.

## Introduction

The novel and virulent coronavirus disease 2019 (COVID-19) virus was discovered in China in November 2019, spread rapidly around the globe, and became a worldwide pandemic storm (Ning et al., 2020). Suddenly, thousands of lives were lost globally, and hospital supplies used to protect health care workers (HCWs) and treat patients dwindled as the number of infections rose. In a bid to flatten the curve of rising infections and reduce the mortality rate, the governments of countries instituted lockdown measures. The lockdown measures entailed restricted or no movement to reduce contact and spread of COVID-19. In addition to the lockdown measures, public health agencies such as the WHO and Centers for Disease Control and Prevention (CDC) developed guidelines and policies that would prevent infections from rising and for the management of available hospital resources (Cdcgov, 2021). One of such policy is the non-hospital visitation policy (Weiner et al., 2021).

### South Africa and COVID-19 Pandemic

Pre-advent of the COVID-19 pandemic, hospital visitation policies have resulted in changes between restricted visitation- and non-restricted hospital visitation. Some health care facilities such as old-age homes adopt open non-restricted visitation policies, while others have restricted policies such as time of visitation and number of people allowed to visit. In South Africa, many in-patient health care facilities adopt the restrictive visitation policy. Visitors are often restricted by numbers and time frame (Appalsamy and McKerrow, 2016). However, during the COVID-19 pandemic, health care systems in South Africa and around the world adopted restrictive visitation in a bid to reduce the death rates from infections and manage available hospital resources (McQuoid-Mason, 2020; Weiner et al., 2021; Zeh et al., 2020). Physical hospital visitation was limited to digital visitation (Morley et al., 2020a; Moolla et al., 2020). The restrictive visitation policies during the pandemic though had the advantage of reducing cross-infection from patients creating unwanted feelings such as anxiety, feelings of loneliness, and not being there for their loved ones. Patients come from families who view the visitation of a loved one as the core of Ubuntu philosophy.

### Restrictive Visitation Policy and Ubuntu Philosophy

The Ubuntu philosophy is an African philosophy that emphasizes caring for one another and cohesiveness in the community (Nolte and Downing, 2019). The Ubuntu philosophy emanates from an idiom that says a person is a person through other persons (Ewuoso and Hall, 2019). The philosophy is part of the socialization of the majority of African children, including Nurses who are part of those communities. The philosophy has also been embedded as part of the ethics of caring in the nursing profession (Mulaudzi et al., 2018).

In South Africa, the cancelling of hospital visitation was a setback to the practice of the African philosophy of Ubuntu. It affected patients, families and nurses. Nurses play a very important role in ensuring a conducive environment for patients to enjoy their stay and have a speedy recovery. Caregiving is a prominent concept in the Ubuntu philosophy which is exhibited in caring and visitation of the sick. (Chisale, 2018; Makhele and Mulaudzi, 2012). Ubuntu centered care revolves around people caring for each other and members of the community looking out for each other during sickness (Banda and Mudzanire, 2019). Therefore, family and community members long to visit a COVID-19 infected patient who is admitted to the hospital. This visitation also helped the family to assess how their family member is recovering. It also offered family members the opportunity to tell their loved ones about the treatment they receive in an institution. Lack of visitation added to the anxiety of not being able to see the progress of their patients, thus casting doubt on what could be happening to their loved ones (Raphael et al., 2021). Patients too looked forward to the family member's visits (during visiting hours) and saw this as part of caring and Ubuntu. The visits make them feel loved and cared for. They are allowed to have conversations with their loved ones. Nurses also enjoy the visiting hours as they also get the opportunity to meet their patients' loved ones. However, the limitation of physical hospital visitation to virtual visitation brought a disequilibrium to the balance brought by Ubuntu to the multidimensional nursing care and the support system of the patients (Siddiqi, 2020).

Studies have documented the impact of the non-visitation policies on patients and their families (Kandori et al., 2020; Raphael et al., 2021) and questioned its lawfulness in the case of children (McQuoid-Mason, 2020), but none have documented the experience of nurses, patients, and families in terms of the African philosophy of Ubuntu on restrictive visitation policy during the pandemic.

## Aim

This paper explored the experiences of caregivers and care receivers in terms of the African philosophy of Ubuntu and the restrictive visitation policy during the pandemic.

## Methods

The study is a qualitative descriptive study. The researchers utilized a narrative inquiry as a means of data collection. The use of webinars as a method of data collection is relatively new in research studies. It has been used by Tiong and Sim (2020) to obtain data to corroborate their quantitative study. We conducted a webinar on moral anguish of caring in the COVID-19 pandemic: the Ubuntu perspective as part of monthly seminar series to enable the key informants to narrate their own experiences.

### Participants

The population comprises caregivers and care receivers in the COVID-19 pandemic. The participants were purposively selected based on criterion sampling. Criterion sampling is used when researchers choose participants based on predetermined criteria. The participants were chosen based on their roles as either a caregiver or care receiver. A total of five presenters were engaged in the dialogue on the impact of restrictive visitation policy during the lockdown. They shared their experiences and what it meant to the Ubuntu culture in which they were socialized. The participants included one intensive care unit (ICU) nurse who was a part of the hospital who was part of those caring for patients admitted in the hospital; two patients, one that was nursed at home and the other fully in the ICU; a family member whose siblings were hospitalized; and the nursing manager who is among those making the decisions at the hospital. The added value in this method is the opportunity of the listeners who often write questions on the chats and ask questions to the participants as well as making comments on their own experience which added more value to this study.

### Data Collection

#### Setting

The data was collected during a webinar that was conducted in a studio using Zoom and aired live on YouTube. The webinar was done after the peak of the second wave in April 2021. Three of the participants were live in the studio, and two joined in the conversation *via* Zoom.

#### Ethical Consideration

The study received ethical approval from the University of Pretoria Ethics Committee 297/2020. Each participant had the opportunity to continue or decline the study after a thorough explanation of the study. Each participant gave verbal consent to the study. The participants were not forced nor lured to participate in the study. Privacy was waived as participants in the studio could be seen and names were used to identify those who joined the webinar through Zoom.

#### Data Collection Methods

Data were collected using a round-table dialogue on a hybrid of studio and webinar discussion. Three of the participants were on the set in the studio with the moderator who is one of the researchers. The webinar took place on March 31, 2021. The program had a total number of 280 views. Viewers were allowed to make contributions and ask questions during the discussion. Before the day of discussion, the program was explained to the participants by the other researcher, and informed consent was obtained. Each presenter who was also the participant was granted the choice to continue or opt out of the program on the day of the program. Each presenter presented their experience. The general question was:

“Discuss your experience as a caregiver or patient of the restrictive visitation policy during the COVID-19 pandemic.”

Probing questions were asked to elicit more information from the presenters.

#### Data Analysis

The discussions of the participants on the webinar were transcribed verbatim. The transcripts were then analyzed using Braun and Clarke (2019) reflexive thematic analysis method. This entails the researchers reading the transcripts and then repeating the reading of the transcripts. By reading the transcripts the researchers could identify the similarities and patterns from the narratives of the participants. The researchers reflected experiences of the participants and grouped the similarities and patterns into themes. From the reflexive thoughts of the researchers, three themes emerged.

## Findings

The themes that emerged from the narratives of the participants are 1) moral anguish of the caregivers, 2) mental health instability, and 3) erosion of trust in health care practitioners.

Each theme would be supported with narratives from the participants. The participants will be identified with pseudo names.

### Moral Anguish of the Caregivers

From the narratives, the caregivers were not just the health practitioners in the health care facilities but also refers to the family members taking care of the patients at home. Both the health practitioners and the family caregivers experienced frustration due to the restricted visitation policy as it is not accommodative to family members. Family members who embody Ubuntu principles such as cohesiveness, interconnectedness, responsibility, mutual respect, and relational consider it their duty to show affection to family members admitted to the hospital by taking turns to visit them. The policy on lack of visitation to COVID-19 infected patients brought different experiences to the HCWs and the infected patient's families. Though the experiences differ, the narrative below would indicate the anguish experienced by the participants.

Pre-COVID-19 times, the ability of the family caregivers to bring the local (indigenous) food and gifts to their hospitalized patients was part of the common practice. It is viewed in the African culture as an act of caring and the Ubuntu principle of sharing. However, with the restrictive visitation policy, family members are not allowed to come into the hospital. In his words, Macho, one of the study participants, indicated how the restrictive visitation policy affected him. He started by sketching the normal process that he was used to; he said,

*“When one of our family members are in hospital we go by the droves about 10 of us. We will get there with a lot of things that we have brought for him and then we will eat some of them. After that, we will sing and we will pray and then we will leave the hospital.”—Macho.*

He then verbalized how frustrated he felt, which also reflected the family members' anguish. He reflected on the 21 days that he was hospitalized and tried to control his emotions. He reflected and sketched his experience on the loneliness that he felt as follows:

*“But being in hospital now during the COVID-19 pandemic, I mean for 21 days not seeing anyone was a real challenge to me. I also understand that visiting should not be allowed because you can bring the virus in or you can come and visit and get sick so I think it's a real challenge. I longed to see people. I was afraid They also longed to see me.”—Macho*

In his reflection, it was easy to deduce the kind of loneliness he felt and how that brought about fear as desperation in the hospital, which is often viewed as an unknown environment. The restrictive policy posed a dilemma as people felt that they can infect others who would be infected during hospital visitation. Hence, compliance with the policy was the best option.

In Ubuntu, a sense of belonging and togetherness is emphasized. A person is a person through others. Macho was frustrated due to loneliness, and he felt abandoned. At the same time, the family felt they were non-caring and not exhibiting Ubuntu to their loved ones. The narrative of participant Macho was buttressed by the narrative of Khuliso who is a family member. The inability to prepare and share the local delicacy with her sister caused moral anguish as she could not provide her sister with the food she desired due to the non-visitation policy. She verbalized her feelings as follows:

*“Sometimes she will call and say you know I'm craving for this sour porridge and gushe (indigenous vegetable), it's that slippery veg that we like so much. In the hospital, there's no Gushe and the taste is not there. She is craving for the gushe and you wish I could cook the food and give it to her just cook this food and give it to her. She will gain the strength but you can't do that”—Khuliso.*

She further narrated that not being able to practice the family norm of eating with her sister when she or any of her siblings are ill made her feel anguished.

*“The other thing that traumatized me so much as I mentioned that I grew up in a Christian family. My parents used to tell us that if one of us is not okay, go and play in her room, make sure that you take food and go and eat there so that you can stimulate her appetite. Now I can't do that because of this COVID. My parents are no more I feel like I'm no more following the norms and values that I've been taught. It's like I am neglecting her and have deserted my family. I can't be there for her*.”*—*Khuliso.

Khuliso reflected on the principle of being relational by sharing food and being there for one another. In this case it was impossible to fulfil, and she attempted to simulate this with a video call between her and her sister, which left both of them even more disturbed rather than being happy.

Tshepo also stated the pain and moral dilemma of not being there for the family when they need you the most. She expressed it in this way:

*“I had very close family members who were admitted due to this pandemic. The one was my sister that I come after. It was my aunt and my grandmother unfortunately the three died. You wish you could be there. You have this faith that you know if I was there I will do something and deep inside you know there's nothing you can do.”—Tshepo*.

Tshepo reflected the feeling of moral distress which was influenced by dealing with the unknown. In Ubuntu caring for another is a form of showing mutual respect and being responsible for another. The inability to be there for her relatives frustrated her and made her feel uneasy. She emphasized that it also affected the process of closure as she did not see them and was not there for them before they passed on.

The moral anguish of Jane who is an HCW stemmed from different aspects. The first is from not having adequate knowledge and understanding about the disease.

The pathophysiology of the disease was different from the known infection with coronaviruses. However, the way the current severe acute respiratory syndrome with the coronavirus 2 (SARS-COV2) progressed rapidly and drastically was challenging and confusing as the pathophysiology was different from other infections.

“*Okay firstly in my career I have never seen patients change conditions so quickly the one moment they will be okay, they will be fine, they will be stable and within the next 24* *hours or so their condition will change drastically, they will be gasping for life. They will be short of breath; they will be in distress. It's taken all of us for a big surprise. You know it has been quite nerve-wracking to see these types of changes because it's something we as nurses are not used to. It's something new to us as well*.”*—*Jane.

Jane reflected her moral anguish which was influenced by the drastic changes of the patient's condition which made her feel like everything was happening so fast. She also was confused as she was expected to report the condition either to the family or the doctor.

Macho also reiterated that the issue of drastic changes was experienced by him stating the rapid change of his condition. He said:

*“I thought I will be there for a few days but my condition changed very quickly and that made me afraid as they wheeled me to ICU. I felt I was doing well and in no time my condition just changed.”—Macho*.

Jane explained that the inability of the patient's family to visit their admitted family member added extra responsibility on the nurses. They had to call family members regularly to update them about the conditions of the patients.

*“When it came to the family specifically in our ICUs we try to establish like a telephone rapport. We could phone the families and tell them listen this is what's happening, the condition has changed as quickly as possible so that they are aware of what's currently happening with the patient.”—Jane*.

In a normal environment, the nurses created a rapport between themselves, their patients and family through the hospital visiting hours. During visiting hours, they can meet the patient's family and discuss his/her condition with them. The families are kept up to date with the patient's condition, and they can also observe if his/her condition is deteriorating.

In this case, nurses felt that their ethical and moral obligations to the patient and family were affected as visitation rights of the family were restricted. The anguish of the nurses was further deepened with the fact that they did not have accurate information to give to the family when they eventually call because they truly did not know. The nurses were used to being able to accurately report the current situation of the patient, but with the COVID-19 it seemed different.

*“Now it's COVID you can't call that family in and say listen, come and see the patient physically. See what we are doing. What we are struggling with and what we are talking about and what are the dangers and what are we foreseeing. They can't see (the patient's family). It's totally abnormal and it causes a lot of distress.”—Jane*.

Amy narrated her story on how the restrictive visitation policy affected the practice in pediatric ward. Under normal circumstances, women who bring their babies to be admitted to the hospital and cannot afford daily transportation are offered lodging premises to enable them to breastfeed their babies. The practice of lodging mothers is often done in most hospitals in South Africa and is seen as an act of the Ubuntu caring philosophy. Amy narrated why they could not allow visitation and lodging mothers and how the separation of the mothers from the babies caused her distress.

*“As a manager taking into consideration my specialty as a pediatric nurse and that's where I have been working with babies and mothers at the same time. We have got a larger facility. When COVID-19 came we also had to release the mothers not to be part of the caring.”—Amy*.

She explained that it was an ethical dilemma. It was difficult to decide on not admitting mothers as their admission may affect treatments and recovery of the babies as well as spreading the COVID-19 virus.

*“At the beginning, we tried to say let us keep the mother lodges as long as we check them daily. It happened that one of the mothers started to have symptoms and later tested positive we checked the other mothers and more of them tested positive. We were left with no option but to release all the mothers and close the large facility. This became the first pain to see a baby in a hospital without a mom, without anybody visiting because it was now too difficult and that was still in the first wave.”—Amy*

Amy's reflection indicates how she experienced moral anguish of letting mothers go and leave their babies unattended. One can also reflect on the pain experienced by the mothers when their babies were admitted to the hospital. They felt that they are not being responsible and the interconnectedness with their babies is being negatively affected.

### Mental Health Instability

When patients are admitted to the hospital for physical illness their mental health is often supported by the visiting of family members to the hospital. The restrictive visitation policy affected the mental status of the HCWs, the patients, and their family members.

Khuliso shared how the policy created anxiety among the family members.

“*I started understanding what Shakespeare always say that cowards die many times before their death. This pandemic changed my character. I became a coward because each time I received a call I would start to shake. Each time I call and my sister don't respond I felt like I was dying. I would start to cry. Later she will respond with a message to say I'm still alive but the message was too short as I'm used to talking to her using these long messages.”—Khuliso*

She expressed the anxiety experienced with the term “I practically died numerous times” because of the anticipated news from the hospital and not knowing what to expect.

*“So, we died many times.”—Kuhliso*

The anxiety affected her communication with her siblings and her daily activities.

*“Another frustrating thing is you become useless. You become powerless. You know you don't even want to see what tomorrow holds for you because to you it's death, death so immediate and you can't sleep. It is so frustrating when things are okay you always worry. when other siblings don't call you are worried why are they not calling? Are they okay? But during the moment you see a call from your sibling, what's this you won't even hold the phone. You think now there's a problem. It means just normal you start crying. ‘You die every day.’ Physically I was fine but spiritually, mentally, psychologically I died because of this pandemic because I couldn't reach out to most of the people that I love so much.”—*Khuliso.

Amy expressed how the restrictive visitation policy led to loss of Ubuntu values and mental exhaustion for the nurses. She stated the following:

*“COVID denied us with Ubuntu, it denied us of having people that come in to talk to the nurses and help the nurses. Patient are not able to talk to the relatives then it becomes more of a pain to the nurses. You know the way COVID changes, a patient that you told the relative yesterday that she's doing well she's improving; her condition just changes rapidly and she dies. And now you are the one to follow in with the relatives to say unfortunately the person I told you about yesterday whose condition was improving, she's no more or he's no more. It becomes a very painful part to the nurses. Also, you must know that when it happens, it is not only one person who dies. After this person dies, the next person dies, the next person dies you end up with three, four people that die at the same time. All these happening within 30 minutes before even you work through your emotions.”—Amy*.

The reflections show how stressful it was to the nursing staff who were nursing these patients. They endured a lot of emotional distress that affected them mentally.

The restrictive visitation policy was not limited to the hospital settings but also to patients who were quarantined or isolated at home. This led to feelings of loneliness even in the comfort of your own home. Amy, who was isolated at home, said the following:

*“When my sister had to buy all the groceries for me because of my isolation, she said you know your yard is a hot spot I'm going to drop everything at the gate and let your daughter come and take them from the gate. I'm going to drive back. It is painful because you're sitting there you wish your sister can come in, even if it is from the window but she felt No! No! No! No! No you are, your whole compound is a hot spot I will not come in but, I brought everything that you need in the house.”—Amy*.

Even though she was at home where you feel your loved ones will come, they still didn't come because of the fear that they would be infected with the virus. Although she is aware that they love her, she understood that they are trying to do the little that they can by bringing her food.

However, fear paralyzed them from practicing the principle of Ubuntu which promotes the value of interconnectedness, relational, and mutual respect.

Tshepo who is also a mother of four was also isolated at home. She corroborated the narratives of Amy on how lonely and frustrated she felt when she couldn't discharge her duties as a mother.

*“On the 17th was my birthday and as a norm, we sing we shower one another with blessings. They had to sing to me outside the door of my room, that is when the twins started to think and realized something is not right with mommy. She's hiding something. They were distressed because they wanted to hug mummy, to kiss mommy. Unfortunately, they were restricted from doing that. I was like I'm losing it but as an ICU nurse, and as a spiritual person, I say no, God has not given me a spirit of fear but a sound mind.”—Tshepo*.

Patients or family members of patients who had been hospitalized from COVID-19 felt isolated because of stigmatization and did not experience the interconnectedness and sense of belonging that they expected from the community. The restrictive visitation policy fueled the stigma. Community members assumed that passing by someone who recovered from the virus would make them ill. This affected the long existence virtue of togetherness, reciprocity, and interconnectedness that existed in the community. People stigmatized patients, and this further deepens the feelings of isolation and loneliness. Khuliso said

*“There was also the issue of stigmatization of a COVID-19 patient and family. lf you have been sick with the condition, when you go back to the community everyone doesn't want to come next to you. They felt like you are going to spread the infection. If there was a death that was related to COVID-19, it was also a very big stigma that says ‘Oh you don't go in that house.’ Yeah, they don't even pass next to the person. I think it must be addressed as a community that you know this is a disease just like any other disease. We cannot be stigmatizing it.”—Khuliso*.

Frustration was another result of the restrictive visitation policy that affected the mental stability of patients and their families. Amy narrated her frustrations when she was only allowed to visit the relative that was about to die. She said the following:

*“If we can allow at least one person to come in it's really very important. There was a relative admitted at the medic clinic they would allow at least one person to come in. Although I questioned it because they only allowed you to come in when they see that the patient is about to pass on. So why was I not allowed to see her when she still can talk but I can only be given an opportunity at the end.”—Amy*.

Furthermore, Khuliso lamented the fact that she wanted to talk to the patient while the patient was still alive. This goes against the caring ethics of Ubuntu, the compassion that a person is to show to the sick person. Unfortunately, the participant is unable to show compassion to the patient. She feels the patient might have felt hopeless and could not fight because no one loved her. Khuliso stated the following:

*“You are given PPE now to look at this person she has passed on. What is the rationale because I wanted to see this person and talk to this person. May be hearing my voice could have helped the person to start fighting?”—Khuliso*.

In addition to the lamentations of Khuliso, Jane, as an HCW, said in the narrative below that the families would not have closure.

*“When it comes to the grieving process, it's difficult because you have to have some form of closure. How do you have closure if you haven't physically seen your family member? I only call you over the phone to tell you, your family member has passed on or not doing well. How do you properly grieve for that patient?”—*Jane.

The researchers reflected that the grieving period with families would take longer because they were not able to visit and in some cases were not able even to view the corpse.

Adding to the frustration and lack of closure is the inability to participate in family funerals. According to Ubuntu, during funerals, there are always issues of solidarity and cohesiveness. The principle followed is that an injury to one is an injury to all. Therefore, families stick together and work as a team during the preparation of funerals. Khuliso was sad when the aunt died and she was not able to visit the grandmother to help with the preparation for the burial. She said the following:

“*Going back to my aunts that were admitted. One died and the other one survived. The one that survived had to prepare funerals. When things were okay, my grandmother will always tell us that whenever there is a function my grandchildren must come and sing. My aunt will call and say she's normal but she will say you can't come into the house. Can you imagine we needed to support my aunt but we can't go there? That was another thing that was so frustrating.”—Khuliso.*

There was also a policy in place in the preparation of the corpse of infected patients. It entailed covering the corpse fully with plastics to avoid infections, and that prohibited the families from paying their last respects and having a ritual for their loved ones.

### Erosion of Trust in Health Care Practitioners

From the participants' narratives, the non-visitation policy caused a lack of trust in the HCW. Patients were not sure of the reports they received from the health practitioners. For instance, Khuliso was not sure of the report of the health practitioner about the sister that was admitted to the hospital. She doubted the messages she received from her sister. The HCW's integrity was questioned. The inability to visit the sister to know what is happening with her made her doubt if the HCWs were telling the truth about the sister being alive. She said the following:

*“I started doubting if she is the one who wrote this message or these are the nurses they're still waiting for the right time to tell us that she is dead.”—Khuliso*.

Ubuntu values expect transparency and integrity from everyone. However, with the rapid changing of the physiology of COVID-10 infected patients, the transparency is questioned. In Khuliso's narratives the community believed that when a COVID-19 patient is admitted to the hospital, the patient would not come out alive. The news received from the HCW about your patient changes so rapidly that you doubt if they were telling the truth or they were lying. Khuliso said the following:

*“You are stuck at the denial like you were saying today you are told that you know the patient has improved so much and you are so happy and then tomorrow we are told no she passed on. Then you start asking yourself questions. Someone said it seems like hospitals are killing patients because that's what people feel or know. If you say a person has improved and then tomorrow you say you know the condition has deteriorated. In some cases, you were not even told that the condition has deteriorated and you are just told that the person has passed on. How do you deal with the issue of acceptance?”—*Khuliso.

Jane believes that the inability of the patients to see the nurses may have contributed to the lack of trust. She said the following:

*“Imagine treating a patient. He doesn't know what you look like. The physical touch and the caring are there but it's in a suit and it's with gloves. They can't see you so how do you trust someone that you can't physically see? The patient would lie in bed and only recognize that the nurses have changed shifts only through the voices. The personalization of everything that we are used to is gone and the fact that the rapport that you build with the families and with the patients is not normal. It's totally abnormal and it causes a lot of distress.”—Jane*.

The reflections painted a picture of distrust that occurs between HCWs, patients, and family members. Family members felt like they were not given the right information. They were desperate to be part of the care of the patients but the policy restricted them. The HCW also felt helpless as they could not communicate with the family members and provide care the way they desired.

### Discussion of Findings

Restrictive visitation policies in a health care facility have been preferred to open visitation policy, which occurs during infectious epidemics or pandemics due to the established benefits of reducing the spread of infections (Garrouste-Orgeas et al., 2016; Brauchle et al., 2021; Moss et al., 2021). However, it has also been associated with unintended results every time it is implemented (Raphael et al., 2021).

In our study, reflections on the concomitants of the restrictive visitation policy during the COVID-19 pandemic showed that the African philosophy of Ubuntu, which was normally present in hospital settings before the outbreak of COVID-19, diminished with the restricted visitation policy. The Ubuntu African philosophy is an important concept in caring that is acknowledged and practiced in the health care facilities in the African culture. It emphasizes values such as reciprocity, responsibility, mutual respect, interconnectedness, and relational in care. Our study showed that the inability to practice these Ubuntu values led to moral anguish, mental instability, and erosion of trust in the health practitioners.

Patients, family members, and HCWs understand the Ubuntu philosophy as the core of caring. They value the crux of Ubuntu by striving to live and practice caring according to the saying and principle of a “… *person is a person only through the other person*…” (Ewuoso and Hall, 2019: 94).

Caregiving in Africa is an act of shared responsibility, and visiting the sick is regarded as an exhibition of Ubuntu value (Banda and Mudzanire, 2019). The family of the sick are able to fulfil their own share of the responsibility of caring for the patient by bringing food and providing emotional and physical support, while nurses also add the same principle of Ubuntu in addition to their professional duties to the sick in their care. The inability to fulfil this responsibility due to the restrictive visitation policy led to moral anguish among patients, family members, and HCWs. Similarly, studies conducted during the COVID-19 pandemic revealed that caregivers experienced moral distress or anguish as they were unable to carry out duties or responsibility as a result of the restrictive policy in place (Morley et al., 2020b; Kandori et al., 2020). The restrictive policy did not only bring moral distress to the caregivers (nurses and family members) but also brought disconnection between the patients, family members, and nurses. As a result of the shared responsibilities before the COVID-19 pandemic, there was an interconnectedness between the nurses, the patient, and the family members.

As each person dispels his/her responsibility, there is an act of mutual respect and interconnectedness that develops. Interconnectedness in Ubuntu emphasizes that an individual cannot exist alone, but that one only exists as we relate and connect with one another. Interconnectedness in Ubuntu values of being together became lost in social isolation and social distancing, the underlining principle of the restrictive visitation policy (Wilder-Smith and Freedman, 2020).

The patients in our study felt forgotten and lonely in the hospital. Families could not work together towards burial rites and rituals of the deceased as entrenched in the values of Ubuntu (Baloyi and Makobe-Rabothata, 2014).

The disruption of the interconnectedness by the changing policy during the pandemic led to a change in care and communication skills with patients and their family members (Morley et al., 2020a). This led to mental exhaustion for nurses, patients, and their families (Zhang W. R. et al., 2020; Zhang Y. et al., 2020).

According to Zhang et al. (2020), the world is experiencing mental exhaustion in the form of depression, loneliness, and mental fatigue which is similar to the experience of our participants. Mental exhaustion can lead to incorrect judgment among HCWs, undermining their decision-making process (Chersich et al., 2020). The values of mutual respect and trust in the Ubuntu philosophy was lost in the pandemic as participants perceived that the judgement of the nurses was inaccurate. Unfortunately, nurses themselves were perplexed at the rapid changes of the COVID-19 symptoms.

Nursing care in the African context cannot be separated from Ubuntu philosophies because of their ethical similarities. From our reflections on the narratives of the participants, we recommend that restrictive policy be practiced on a case-by-case application to the benefits of the patients and their family members. Furthermore, in the ambiance of shared responsibility, family members can appoint one person that will visit the patient and bear the cost of the full protective gear that would be worn at each visitation. Furthermore, the visitation hour can be reduced instead of strict non-visitation.

### Implications to Health Care

This issue affects the ethics of care as HCWs are unable to practice and depict caring and Ubuntu values. The inability of the HCWs to exhibit the values and philosophy they have embodied affects the quality of care they provide. Policies that are formulated in hospital settings must be congruent to the needs of the patients, families, and society at large. Failure to incorporate the needs of the public in the policy-making process may affect the compliance of the public to the preventive health measures that are propagated.

### Contributions and Limitations

It is important to note that other researchers reported other matters relating to the restrictive visitation policy. It is equally important to reflect on the effect of the pandemic and the restrictive policy on the African culture and the Ubuntu philosophy. Hence, our study brought out the effect of the restrictive policy during the COVID-19 pandemic and the effect on the African culture and Ubuntu philosophy.

## Data Availability

The raw data supporting the conclusion of this article will be made available by the authors, without undue reservation.
